# Goat Dairy Product Assortment in Different Sales Channels in Northwestern Italy

**DOI:** 10.3390/ani9100823

**Published:** 2019-10-17

**Authors:** Stefano Massaglia, Danielle Borra, Valentina Maria Merlino

**Affiliations:** Department of Agricultural, Forest and Food Sciences, University of Turin, Largo Paolo Braccini 2, 10095 Grugliasco, Italy; stefano.massaglia@unito.it (S.M.); danielle.borra@unito.it (D.B.)

**Keywords:** goat dairy products, sales channel, offer characteristics

## Abstract

**Simple Summary:**

Goat dairy product manufacturing in Piedmont has increased in recent years, along with consumption. This research highlights the characteristics of goat cheese, milk, and yogurt available in three categories of sales channels, large-scale retail, retail sales, and farmers’ markets, in a metropolitan area in Northwestern Italy. We reveal clear differences between the offered products’ characteristics in the various distribution channels, both in terms of the reference numbers and assortment, and in terms of price, quality, and typicality. In particular, the analysis of product origins shows a correlation between the share of local product presence and the distribution channel type. Given the importance of product origin for consumers, as well as consumer acceptance of goat cheeses, the increase of local goat dairy products in farmers’ markets or retail outlets could represent an opportunity for growth and differentiation of this sector in the regional market, allowing operators in the sector to benefit from direct sales advantages.

**Abstract:**

An analysis of goat dairy-based product assortment was carried out in the metropolitan area of Turin (Northwestern Italy), considering three different sales channels: large-scale retail chains, retail stores, and direct sales in farmers’ markets (FMs). The survey results show a widespread presence of goat products in the Turin market. In each type of selected distribution channel, characterized by its own peculiarities, products differed both in terms of reference numbers and assortment; they were better in large-scale retail distribution, both in terms of quality and typicality, whereas specialized retail and direct sales were better equipped. Furthermore, given the importance of the sector at the regional level, we also focused, through the analysis of product origin, on the fact that local provenience increases from large-scale distribution to fully regional farmers’ markets. The mean price was different, being lower in direct sales at FMs, and medium to high and high at retail sales, as they are considered high quality niche products. The offer is correlated and in agreement with consumer targets (modern, ethical, and traditional), finding the "ideal" product in the different types offered, however, even if the results underline the potential of this sector, the fragmentation of the goat sector in Piedmont still represents a limit to expansion, and to the positioning of products in the market.

## 1. Introduction

Europe holds only 1.9% of the world’s goat population, however, the Member States together have achieved worldwide production levels of 15.1% milk and 35.1% goat cheese [1]. At the community level, the milk sector consists of 96.7% cow’s milk, 1.8% sheep milk, and only 1.5% goat milk, however, the goat species plays a fundamental socioeconomic and environmental role in many regions of Europe, including in Italy. In fact, the national production of goat milk has increased in recent years, from 11,840 tons in 2010 to 31,000 tons in 2016 (+162%) and 70% of the total production is for cheese making [2,3]. The amount of goat cheese produced nationwide has increased from 4540 to 6530 thousand tons in a five year period (2013–2017) and at the same time the dairy goat production industry has developed by differentiating commercial offers according to an increase in consumption [4,5]. Until recently, goat products, cheese in particular, were considered essentially niche products, but in recent years they seem to have gradually assumed the characteristics of mass consumption products. In fact, the evolution of consumption styles oriented towards the search for health-promoting products for consumers corresponds to the features of goat dairy products, which are lower in fat (in the case of cheeses, fat globules, smaller than those present in cow’s milk, escape the casein network during cheesemaking), more digestible, and rich in essential fatty acids, in comparison to cow dairy products [5,6,7,8,9]. In addition, even if goat cheeses represent a heterogeneous product category, they share peculiar flavors and aromas appreciated by consumers [10,11]. 

In Güney (2019) [5], it emerged that price, health and nutritional values, digestibility, and availability were the most important factors affecting consumers during consumption and purchase of goat milk and its products. The same study described consumers as mostly preferring cheese from among the various goat dairy products, and they mainly used supermarkets as the place of purchase. Therefore, the product availability and assortment in the market play a key role in determining consumer choice during the purchasing process. In the national context, in addition to traditional sellers (specialized cheese shops and delicatessens), it is currently easy to find different types of goat dairy products, as well as milk, in many other distribution channels, however, the distinctive characteristics that define the sales channel categories (large-scale retail chains versus retail sales or retail sales versus farmers’ market) affect both product type availability and quality standard characteristics of the products [12,13]. The large-scale retail chain structure logistics and specifications, for example, enable them to guarantee, long term, consistent quality standards, as well as the availability of the products [14]. Direct sales of goat cheeses by producers in farmers’ markets are generally based on traditional products, linked to the territory which are of high sensory quality, however, they are closely linked to the seasonality of goat production managed at the family level. In fact, dairy goat farming systems in the Italian context are characterized by high variability, from intensive indoor, highly productive and specialized breed production, to semi-extensive and extensive outdoor systems with seasonal production, local breeds, and fluctuating quality trends [15]. This research outlined the assortment of cheeses and other goat products (milk, yogurt, and butter) and analyzed different sales channel types distributed in the metropolitan area of Turin in northern Italy. Specifically, goat product characteristics at several points of large-scale retail chains, specialized shops, and farmers’ markets have been analyzed. The research aim was to identify commodity-related product characteristics and potential differences between the analyzed sales chains. Finally, the presence of local producers within commercial offers was evaluated.

## 2. Materials and Methods 

In order to describe the goat dairy product assortment in the metropolitan area of Turin, the offers at different sales points of large-scale retail chains (LRC), retail sales (R) (specialized cheese shops and delicatessens), and producers at farmers’ markets (FM) were analyzed in two periods during 2016, one period from January to March and the second period from April to June. A large-scale retail chain refers to a group of sales outlets bearing one or more common commercial signs, organized over large areas, and generally belonging to an organization or group. The points of sale of LRCs are classified by the size and number of available references [16]. In particular, hypermarkets are structures with a retail area of more than 2500 m^2^ and with a number of references generally between 7000 and 40,000; a supermarket is defined by a sales area of 400 m^2^ to 2500 m^2^ and a number of references offered between 5000 and 10,000 items; discounters are points of sale where the assortment does not provide for the presence of branded products in an area generally between 200 and 1000 m^2^ and with a number of references usually less than 1000; and finally, a free service means a small structure (local store), generally connected to a large brand, with a retail area ranging from 100 m^2^ to 400 m^2^. 

At the retail sales (specialized stores), generally located within the city, usually the retailer establishes a direct relationship with the buyers. The retailer buys large quantities of goods from the producer or wholesaler and sells small quantities to the end customer. The size of this store is small, however, the references and turnover of these activities depend closely on the capabilities of the retailer, and the quality and price of the products sold [17,18]. 

Finally, farmers’ markets could be seen as historical markets; the flagship of local food systems [19]. This alternative food network is an instrument for the recovery and communication of knowledge linked to the traditions and territory of production. At the same time, it enables a relationship of trust to be established between the consumer and the seller [20]. 

Data collection was directly undertaken using a checklist and, where possible, by means of face-to-face interviews with sales staff. The collected information at each point is shown in Table 1.

Because the point of sale size and typology influence the assortment, positioning, quality, and format of sales products [15,18,21], in the case of LRC, the main signs of the considered geographical area were selected, considering hypermarkets, supermarkets, free services, and discounters. In addition, product positioning was also recorded (refrigerated counter or wool), as this is directly linked to the product type (ripened, fresh), but also because it includes different positioning strategies, on the shelves or in the refrigerator, which directly affects the product visibility and attractiveness to the buyer [22,23]. 

Concerning the retailer’s category (R), the survey was carried out considering historic specialized shops in the city. In particular, the choice to analyze retailers was made in order to assess the differences in terms of assortment, but also quality, origin and price of the products. In fact, this type of sales point usually provides typical products of higher quality as compared to large-scale retailers, perhaps linked to the territory and niche, and therefore characterized by higher prices [24,25]. The last category of research (FMs) referred to direct sales by producers, which are periodically involved in the city in the *Campagna Amica* markets promoted by Coldiretti (organization of farmers at the national and European level) (https://www.campagnamica.it/). Farmers’ markets have become an increasingly important point of food purchase by consumers because of the purchasing process mechanisms are oriented toward quality, security, and sustainability [26,27]. In addition, this type of commercialization provides opportunities for small family farms to penetrate the market with a small production volume [28]. 

The checklist used in the data collection phase (Table 1) also included descriptive product data, i.e., in addition to the type of product (milk, yoghurt, butter, or cheese), the producer name [23,29], the origin [28,30,31], the price [27,32,33] and the presence of offers [34,35] were recorded. In the case of milk, all types of milks (pasteurized, long-life, raw, skimmed, partially skimmed, and whole) were registered during data collection into the unique category of milk products (due to the limited offers), whereas, in the case of cheese, the distinction between spreader, fresh/ripened, soft/semi-hard/hard, blue, stretched, and grated products was registered. A descriptive analysis (count, mean, and standard deviation) of the goat product assortment was carried out and compared considering each of the selected categories of outlets, with the aim of highlighting the main differences in terms of number, type, price, positioning, and origin of the offered goat products.

## 3. Results

**Figure 1:**
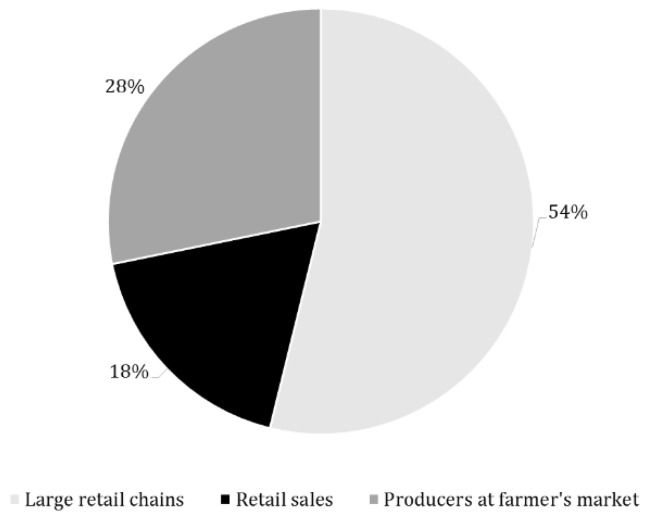
Percentage of goat product points of sale belonging to each sales channel category.

A total of 39 points of sale of goat dairy products were analyzed and their proportion for each considered sales channel category is reported in Figure 1. 

**Figure 2:**
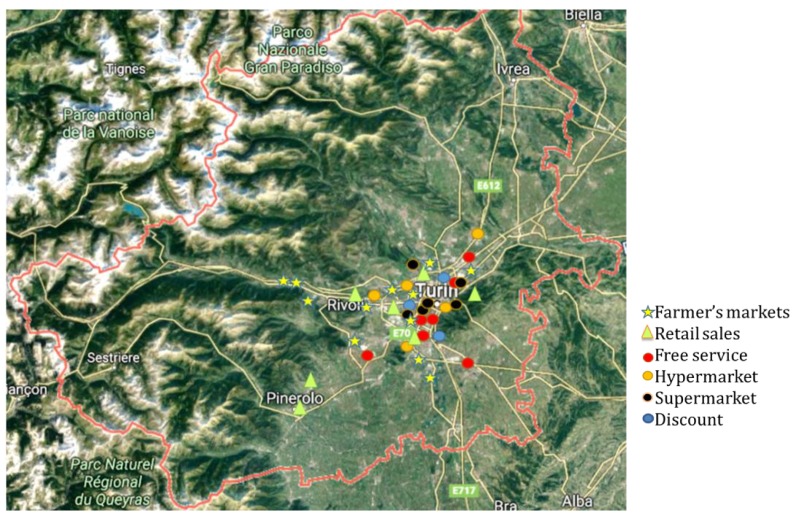
Sampling points distribution in the metropolitan area of Turin.

The sampling distribution in the metropolitan area of Turin (Piedmont, Northwestern Italy) is described in Figure 2. 

Information on 494 goat dairy products was collected at large-scale retail chains at 21 different points of sale among hypermarkets (24%), supermarkets (29%), free services (33%), and discounters (14%). A total of 140 different producers, whose origins are shown in Table 2, satisfied the product supply at LRCs. A total of 80% of the dairy products sold at LRCs were national (Italian origin), of which 33% were regional (Piedmont). Moreover, among Piedmontese producers, a majority were located in the Cuneo district (54.5%). The 80% of foreign products were, on the other hand, mostly of French origin, followed by products made in Austria, Germany, and Greece. 

Regarding retail sales, data on 78 goat dairy products were collected at seven specialized stores. Compared to the references analyzed in the LRC points of sale, the origin of goat products marketed by retail sales was 79% regional (Table 2), with two points of sale specializing in the sale of local products only (Table 3).

Information on 49 goat dairy products were collected at 12 farmers’ markets by interviewing 11 producers belonging to the Turin (85%) and Asti e Novara Piedmontese districts (Table 2). All the analyzed products were made in the regional area. In this sales channel, no producers located in Cuneo district sold their products. The presence of Piedmontese products increased from the large-scale retail (27%) to specialized (79%) and direct (100%) channels.

**Figure 3:**
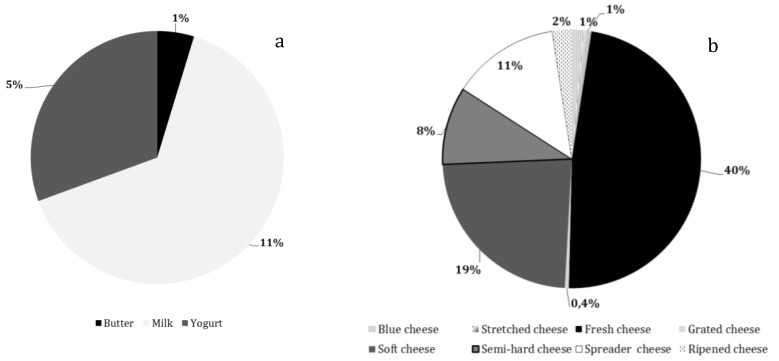
Percentage of references (total number = 494) of goat dairy products offered at large-scale retail chains: (**a**) butter, milk, yogurt and (**b**) cheese references divided by typology (spreader, fresh/ripened, soft/semi-hard/hard, blue, stretched, grated).

Information reported in Figure 3 describes the supply of goat dairy products at LRCs, which consisted mainly of fresh (48%) and soft (23%) cheeses. 

At large-scale retail chains, goat products were almost equally positioned between the lanes (53%) and refrigerated counters (47%) (Table 4), however, by differentiating the products according to their origin, it is clear that foreign products (French) are mainly placed in the lanes, suggesting the presence of ripened and packaged cheeses that do not require refrigeration. On the contrary, Piedmontese products were mainly placed in the refrigerated counter, showing the overwhelming presence of fresh products (fresh cheeses, yoghurt, and spreads).

Considering LRCs, the products offered at the different point of sale types in the retail chain increased with an increase of the sales area (Table 5). The reference numbers by type of point of sale was very variable. In general, hypermarkets offered 20 different goat dairy products as compared with an average number of below one in the case of discount stores. On the contrary, there was no significant difference in the comparison of the LRC types for number of references and producer ratio. On average, each producer sold 1.6 products through large-scale retail trade. Goat dairy products had an average price of 16.39 €/Kg, with a maximum average price of 18.28 €/Kg in free service and an average minimum price of 14.43 €/Kg recorded in discounters. 

**Figure 4:**
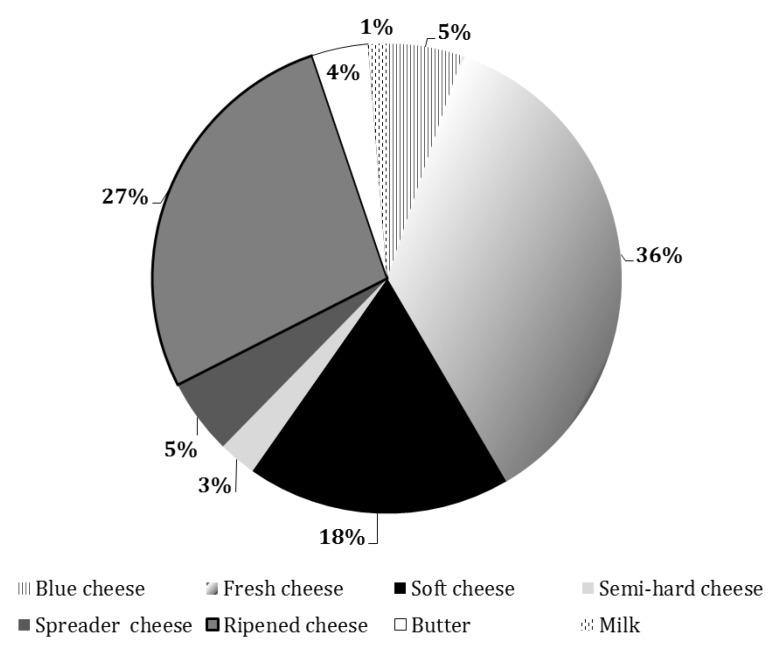
Percentage of references (total number = 78) of goat dairy products offered at retail sales shops, divided by typology.

The analysis of product typologies (total number = 78) offered at specialized shops is described in Figure 4.

At specialized stores and retailers, almost all of the offered goat products were cheeses, especially fresh (36%), ripened (27%), and soft (18%) (Figure 4). Therefore, the assortment was more categorized but differentiated within the range of cheeses themselves. Milk was rarely present, confirming its almost exclusive presence in LRC points of sale. 

**Figure 5:**
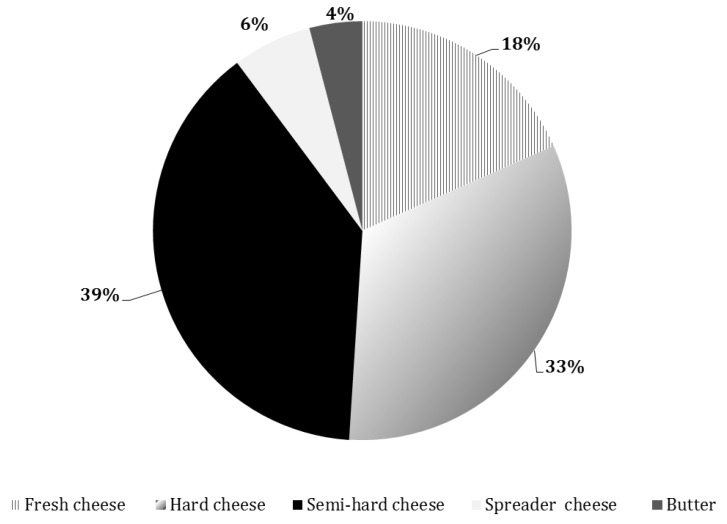
Percentage of references (total number = 49) of goat dairy products offered at farmers’ markets, divided by typology.

The goat product assortment decreased in farmers’ markets by up to 49 dairy products, in comparison to LRC and RC points of sale. Cheeses were the predominant offered products, especially semi-hard ones (Figure 5). Table 6 also shows that some farms (numbers 2 and 3) specialized in the production of a single product, probably due to a dual productive attitude (meat and milk).

**Figure 6:**
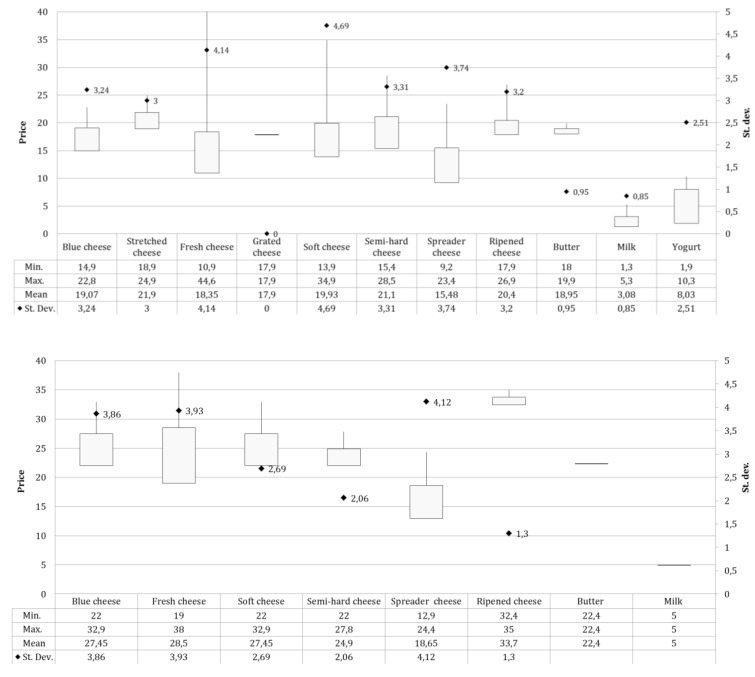

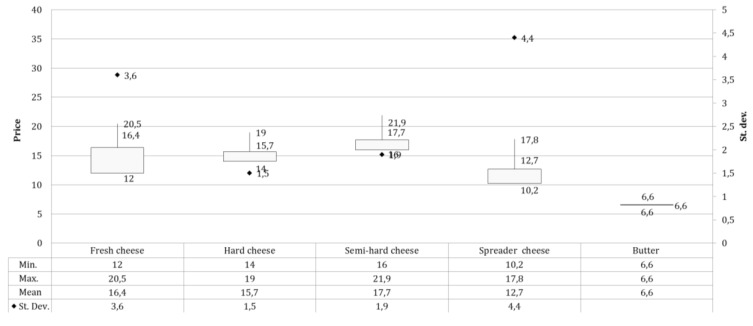
The minimum and maximum values of price (€/Kg), the average price (€/Kg), and the standard deviation are reported for each product type collected at: large-scale retail chains (a), specialized shops (b), and farmers’ markets (c).

The product prices were analyzed for references collected at large-scale retail chains, retail sales, and farmers’ markets, and are reported in Figure 6. 

In general, from the farmers’ market to the LRC, prices increased for soft (+39.4%), spreader (+24.4%), and semi-hard cheeses (+15.8%), whereas for the comparison of large-scale retail chains with retail sales, there were price increases for milk (+60%), spreader (+48.7%), butter (+44.4%), and for fresh and semi-hard cheeses (30.5%). In particular, at large-scale retail chains, the highest average price was recorded for stretched cheeses (21.9 €/kg), followed by semi-hard cheeses (21.1 €/kg). The average price differential between the various types of product, from the lowest average price for spreadable cheeses to the highest for stretched, was equal to € 6.42, however, the number of references of other products than cheeses was important, and in particular milk had an average price of 3.08 €/liter. In retail sales, in parallel to the decrease of the number of references, the average sales price showed a clear increase, with an average value equal to 31.8 (€/kg). For the producers, both the assortment and the average price were characterized by a further reduction (mean selling price 16.70 €/kg). 

## 4. Discussion and Conclusions

The survey results show that the presence of goat products on the Turin market is widespread in all three types of considered sales channels. Each distribution types has its own peculiarities, both in terms of reference numbers and assortment, which are greater in large-scale retail trade, while in terms of quality and typicality the specialized details and direct sales are better equipped [28,36]. The findings suggest that the differences between the analyzed channels allow differentiation of the product offers, to answer the needs of different consumer targets [27,33]. In particular, large-scale retailers offer the widest and most varied range of products. The goat assortment included over-the-counter products, where it is also possible to find high quality products even of mainly industrial origin, and over-the-counter products where it is possible to choose products more oriented to the modem consumer, linked, for example, to the practicality of use (packaged products) [37,38]. Specialized shops respond to consumers who are willing to pay more than the average price at the other types of sales channels for products belonging to producers who have been established for years as the sector’s elite, although they do not have production volumes that allow them to deal with large-scale retailers [39]. Through direct sales at farmers’ markets, the types and origins of the products are in line with the targets of consumers, who are increasingly attentive to the environmental, ethical, and social sustainability linked to traditional production, as well as to the link between products and the local territory [40,41,42,43]. Another difference between the assortment of the different points of sale was the typology of products offered which included: at large-scale retailers there were mainly fresh cheeses, as well as an important percentage of soft cheeses and milk; among the retailers there was a notable percentage of fresh cheeses offered, along with aged cheeses; and at the farmers’ markets 72% of the offer was represented by hard and semi-hard cheeses (often linked to traditional products, and easier to produce in small production facilities as compared with fresh products) [44,45]. With respect to cow dairy products, from the data of Assolatte 2018 [46] it emerges that the supply at the national level (intended as the average number of references by type of distribution channel) in large-scale retail chains is almost double that of goat dairy products, considering hypermarkets, supermarkets and discount stores, however, a greater percentage of industrial and processed products feature which, in the case of goat products, are still scarce or absent in the market. In the same report by Assolatte 2018, references are made to the number of cow’s milk cheeses (national average) available in local shops, which corresponds in part to the number found in our work, however, there is a difference in the type of vaccine products offered. In this case, too, we find a less obvious link with traditional products and products linked to the territory; rather, there is a preponderance of industrial and processed products. 

The analysis of producer origin shows a correlation between the share of local products offered and the distribution channel, highlighting the importance of direct sales for smaller companies located throughout the regional territory. This result is also confirmed in other regions at the national and international level, as it is influenced by characteristics from which advantages and disadvantages are derived, typical of direct sales, as well as market rules applicable globally [15,38,39,47]. The Piedmontese product presence has gradually increased, from LRCs to retail sales to direct sellers, with percentages of 27%, 62%, and 98%, respectively. Cuneo was the district from which the largest number of products originated at large-scale retail traders, whereas for farmers’ markets it was the province of Turin that most reflected direct sales and short supply chains. The short supply chain, in addition to ensuring a sustainable, higher quality, and more secure product [48,49,50], guarantees small producers market access with a seasonal product, even without consistent quality not conforming to the standard required by large-scale retail distribution [38]. The absence of Cuneo producers among those interviewed at farmers’ markets could be related to the structural differentiation and the production systems of goat farms in the different areas. In fact, in the Turin district in particular, goat breeding is characterized by small family-run farms located in the mountain marginal areas that exploit territory resources through semi-extensive systems [51,52]. On the contrary, increasingly widespread intensive breeding of specialized breeds with high productivity in the Cuneo district has been noted [53]. To better define the results regarding producers belonging to the Cuneo district, it would be interesting to analyze the goat dairy supply at farmers’ markets in the Cuneo area. 

As far as prices are concerned, there was a very limited presence of products on offer (5.3% of the total). The direct sales channel has the lowest average values. In this case, the absence of intermediaries (a short supply-chain) and the low business costs for sales structures allow more competitive prices for dairy products with respect to RS mean prices [41,54,55]. On the one hand, the new generations of producers at farmers’ markets are constantly seeking a balance between tradition and innovation, both for the quality guarantee of the product and for the producer’s quality of life, including production costs [55,56]. On the other hand, the specialized retail channel represents the channel with the highest prices, although it is of high quality. Large-scale retailing is at an intermediate level in terms of the price of the offer, thanks to the advantageous organizational features that characterize it [20,38]. This last result is also confirmed for cow’s milk cheeses, for which the best quality/price ratio is in large-scale distribution, where, moreover, the offer is often enhanced by the promotion of prices [32,34,35]. Price competitiveness in farmers’ markets remains high, also for other products (cow’s or sheep cheese), in accordance with price policies that do not see intermediaries intervening in the price increase, unless it is the sale of niche products or provision of special production systems [55,57].

In conclusion, this study provided information on goat product characteristics in the analyzed geographical area. A limitation of the research was the investigation area and, in future work, it should be extended to other areas of Italy where goat farming is widespread (the south and major islands). Nevertheless, this research highlighted that these products in the metropolitan area of Turin in the three analyzed distribution channels can still have good potential for growth and development. In particular, the promotion of other products such as yogurt, which is a product much sought after by consumers, could be an opportunity for producer development. Although goat products have had a negative reputation for many years for their "strong, smelly, salty or sweet" organoleptic characteristics [58], today it can be said that they play an important role, although it is one still overtaken by the predominant role of cow’s milk cheeses, on the market. Goat products have the inherent values of being sustainable, traditional, and healthy in the eyes of the consumer. In fact, the concept of the quality of these products has recently evolved considerably. Animal welfare, the farming environment, and the production organization are now considered to a greater level [56]. It is clear that the offer type is related to and in agreement with the consumers’ target (modern, ethical, and traditional), which finds in the different types of offers its ideal product, however, the fragmentation of the goat production sector in Piedmont still represents a limit for expansion and positioning of products in the market.

## Figures and Tables

**Table 1:** Information collected regarding the goat dairy products sold at each analyzed point of sales.

General Information	Product Information
Type of sales channel (LRC, R, FM) In case of LRC: hypermarket, supermarket, free service or discount Sign Place Date Positioning (refrigerated counter o lane).	type of product (milk^1^, yogurt, butter, cheese); in case of cheese: spreader, fresh/ripened, soft/semi-hard/hard, blue, stretched, grated; producer origin price offer (if present).

^1^ All the milk types (pasteurized, long-life, raw, skimmed, partially skimmed, and whole) collected during data collection were considered in a unique category of milk product.

**Table 2:** Country and region of origin (in the case of Italian products) of the goat dairy products collected in the three categories of sales channels. The district of origin is also reported for Piedmontese products.

Origin	References (Number)		
	Large-Scale Retail Chains	Retail sales	Farmers’ Market
Italy			
Piedmont	130	62	49
District			
Turin	21	30	38
Cuneo	56	21	
Asti	22	2	2
Biella	22	4	
Novara	2	5	9
Alexandria	7		
Lombardy	115	1	
Sardinia	92	2	
Lazio	20	1	
Val D’Aosta	6	2	
European Countries			
United Kingdom	31	2	
Belgium	10	1	
France	82	7	
Total	494	78	49

**Table 3:** Details of the number of references (goat dairy products) divided by function of origin (regional or other), collected at the seven specialized shops.

Sign	Regional (Piedmont) Products	Other Origin	Total	% of Regional Products
a	5	6	11	45%
b	9	0	9	100%
c	9	1	10	90%
d	11	4	15	73%
e	13	2	15	87%
f	12	0	12	100%
g	3	3	6	50%
Total	62	16	78	79%

**Table 4:** Goat product positioning at large-scale retail chains in the considered period, by function of the origin indication.

Country of Origin	Refrigerated Counter	Lane	Total
France	12	70	82
Lombardy	53	62	115
Piedmont	92	38	130
Sardinia	49	43	92
Other	25	50	75
Total	231	263	494

**Table 5:** Number of references (goat dairy products), producers, and products sold per producer (no. of references/no. of producers) in the different types of retail chains points of sale.

LRC types	References (no.)			Producers (no.)			No. References/No. Producers	Mean Price (€/kg or €/l)
	Mean	Min	Max	Mean	Min	Max		
Hypermarket	20.2	9	36	11.4	4	17	1.8	15.87
Supermarket	9	1	22	5.7	1	12	1.6	16.99
Free service	7	2	15	5.1	2	9	1.4	18.28
Discount	0.7	0	2	0.7	0	2	1	14.43

**Table 6:** Detail of the number and types of products recorded at farmers’ markets and sold by some of the involved producers.

Product type	Producer					Total
	1	2	3	4	5	
Cheeses						
Fresh	6	1	-	-	2	9
Soft	4	-	-	6	6	16
Semi-hard	14	-	-	3	2	19
Spreader	2	-	1	-	-	3
Other products						
Custard	2	-	-	-	-	2
Total	28	1	1	9	10	49

## References

[1] FAOSTAT (2019). Livestock Primary. http://www.fao.org/faostat/en/#data/QL/visualize.

[2] ASSOLATTE (2017). Rapporto ASSOLATTE. http://report.assolatte.it/2017/.

[3] ASSOLATTE (2016). Rapporto ASSOLATTE. http://report.assolatte.it/2016/.

[4] Costa M.P., Monteiro M.L.G., Frasao B.S., Silva V.L., Rodrigues B.L., Chiappini C.C., Conte-Junior C.A. (2017). Consumer perception, health information, and instrumental parameters of cupuassu (*Theobroma grandiflorum*) goat milk yogurts. J. Dairy Sci..

[5] Güney O.İ. (2019). Consumer Attitudes towards Goat Milk and Goat Milk Products: A Pilot Survey in South East of Turkey. Turk. J. Agric.-Food Sci. Technol..

[6] Verruck S., Balthazar C.F., Rocha R.S., Silva R., Esmerino E.A., Pimentel T.C., Freitas M.Q., Silva M.C., Prudencio E.S. (2019). Dairy foods and positive impact on the consumer’s health. Adv. Food Nutr. Res..

[7] Ranadheera C.S., Naumovski N., Ajlouni S. (2018). Non-bovine milk products as emerging probiotic carriers: Recent developments and innovations. Curr. Opin. Food Sci..

[8] Szajewska H., Shamir R. (2013). Evidence-Based Research in Pediatric Nutrition.

[9] Turck D. (2013). Cow’s milk and goat’s milk. World Rev. Nutr. Diet..

[10] Mendonça Á., Sousa F., Fernandes A., Carvalho M., Neto I., Gomes S. (2017). Consumers’ perception about sensorial characteristics of the Extra Long Maturation Transmontano Goat Cheese. Rev. De Ciências Agrárias (Port.).

[11] De Barros C.P., Rosenthal A., Walter E.H.M., Deliza R. (2016). Consumers’ attitude and opinion towards different types of fresh cheese: An exploratory study. Food Sci. Technol..

[12] Pearson D., Henryks J., Trott A., Jones P., Parker G., Dumaresq D., Dyball R. (2011). Local food: Understanding consumer motivations in innovative retail formats. Br. Food J..

[13] Aschemann J., Hamm U., Naspetti S., Zanoli R. (2007). The organic market. Org. Farming: Int. Hist..

[14] Lee J., Gereffi G., Beauvais J. (2012). Global value chains and agrifood standards: Challenges and possibilities for smallholders in developing countries. Proc. Natl. Acad. Sci. USA.

[15] González-Benito Ó., Martos-Partal M., Garrido-Morgado Á. (2018). Retail store format decisions. Handbook of Research on Retailing.

[16] (2019). PIANISETTORE. http://www.pianidisettore.it/flex/cm/pages/ServeAttachment.php/L/IT/D/2%252Ff%252F0%252FD.786025e999e6177c2cfb/P/BLOB%3AID%3D1033.

[17] Cox R. (1959). Consumer convenience and the retail structure of cities. J. Mark..

[18] Škubna O., Homol J., Belova A.V. (2017). Domestic and Foreign Origin Foodstuff Prices Comparison in Selected Retail Chains. Agris-Line Pap. Econ. Inf..

[19] Brown C., Miller S. (2008). The Impacts of Local Markets: A Review of Research on Farmers Markets and Community Supported Agriculture (CSA). Am. J. Agric. Econ..

[20] Corsi A., Barbera F., Dansero E., Peano C. (2018). Alternative Food Networks.

[21] Kamran-Disfani O., Mantrala M.K., Izquierdo-Yusta A., Martínez-Ruiz M.P. (2017). The impact of retail store format on the satisfaction-loyalty link: An empirical investigation. J. Bus. Res..

[22] Husen S. (2017). The Mediating Role of Product Positioning Quality and Product Attractiveness Advantage. Int. J. Bus. Manag. Sci..

[23] Aliagha G.U., Qin Y.G., Ali K.N., Abdullah M.N. (2015). Analysis of shopping mall attractiveness and customer loyalty. J. Teknol..

[24] Kvam G.-T., Magnus T., Petter Straete E. (2014). Product strategies for growth in niche food firms. Br. Food J..

[25] Loureiro M.L., Hine S. (2002). Discovering niche markets: A comparison of consumer willingness to pay for local (Colorado grown), organic, and GMO-free products. J. Agric. Appl. Econ..

[26] Trobe H.L. (2001). Farmers’ markets: Consuming local rural produce. Int. J. Consum. Stud..

[27] Curtis K.R., Salisbury K., Ward R., Durward C. (2019). Targeting Farmers’ Markets in Utah: Understanding Fresh Produce Pricing.

[28] Diamond A., Barham J. Moving Food along the Value Chain: Innovations in Regional Food Distribution. https://ageconsearch.umn.edu/record/145618.

[29] Santos J.F., Ribeiro J.C. Product attribute saliency and region of origin: Some empirical evidence from Portugal. Proceedings of the 2005 International Congress.

[30] Norris A., Cranfield J. (2019). Consumer Preferences for Country-of-Origin Labeling in Protected Markets: Evidence from the Canadian Dairy Market. Appl. Econ. Perspect. Policy.

[31] Bentivoglio D., Savini S., Finco A., Bucci G., Boselli E. (2019). Quality and origin of mountain food products: The new European label as a strategy for sustainable development. J. Mt. Sci..

[32] Smith A., Raven R. (2012). What is protective space? Reconsidering niches in transitions to sustainability. Res. Policy.

[33] Massaglia S., Borra D., Peano C., Sottile F., Merlino V.M. (2019). Consumer Preference Heterogeneity Evaluation in Fruit and Vegetable Purchasing Decisions Using the Best–Worst Approach. Foods.

[34] Elberg A., Gardete P.M., Macera R., Noton C. (2019). Dynamic effects of price promotions: Field evidence, consumer search, and supply-side implications. Quant. Mark. Econ..

[35] Sinha I., Smith M.F. (2000). Consumers’ perceptions of promotional framing of price. Psychol. Mark..

[36] Andrée P., Dibden J., Higgins V., Cocklin C. (2010). Competitive Productivism and Australia’s Emerging ‘Alternative’Agri-food Networks: Producing for farmers’’ markets in Victoria and beyond. Aust. Geogr..

[37] Banerjee S., Joglekar A., Kundle S. (2013). Consumer awareness about convenience food among working and non-working women. Ijsr Int. J. Sci. Res..

[38] Accorsi R., Manzini R., Pini C. (2017). How Logistics Decisions Affect the Environmental Sustainability of Modern Food Supply Chains: A Case Study from an Italian Large-scale Retailer. Sustain. Chall. Agrofood Sect..

[39] Sckokai P., Soregaroli C., Moro D. (2013). Estimating market power by retailers in a dynamic framework: The Italian PDO cheese market. J. Agric. Econ..

[40] Berti G., Mulligan C. (2016). Competitiveness of small farms and innovative food supply chains: The role of food hubs in creating sustainable regional and local food systems. Sustainability.

[41] Colonna A., Durham C., Meunier-Goddik L. (2011). Factors affecting consumers’ preferences for and purchasing decisions regarding pasteurized and raw milk specialty cheeses. J. Dairy Sci..

[42] Gracia A. (2014). Consumers’ preferences for a local food product: A real choice experiment. Empir. Econ..

[43] Blasi E., Cicatiello C., Pancino B., Franco S. (2015). Alternative food chains as a way to embed mountain agriculture in the urban market: The case of Trentino. Agric. Food Econ..

[44] Fontecha J., Peláez C., Juárez M., Requena T., Gómez C., Ramos M. (1990). Biochemical and microbiological characteristics of artisanal hard goat’s cheese. J. Dairy Sci..

[45] Emaldi G.C. (1996). Hygienic quality of dairy products from ewe and goat milk. Proceedings of the Production and utilization of ewe and goat milk.

[46] ASSOLATTE (2018). L’andamento delle vendite dei mercati. http://mercati.assolatte.it/201803/.

[47] Lee C.K.C., Levy D.S., Yap C.S.F. (2015). How does the theory of consumption values contribute to place identity and sustainable consumption?. Int. J. Consum. Stud..

[48] Cornale P., Renna M., Lussiana C., Bigi D., Chessa S., Mimosi A. (2014). The Grey Goat of Lanzo Valleys (Fiurinà): Breed characteristics, genetic diversity, and quantitative-qualitative milk traits. Small Rumin. Res..

[49] Xie K., Wu Y., Xiao J., Hu Q. (2016). Value co-creation between firms and customers: The role of big data-based cooperative assets. Inf. Manag..

[50] Schröck R. (2014). Valuing country of origin and organic claim: A hedonic analysis of cheese purchases of German households. Br. Food J..

[51] Ribeiro A.C., Ribeiro S.D.A. (2010). Specialty products made from goat milk. Small Rumin. Res..

[52] de Asís Ruiz Morales F., Genís J.M.C., Guerrero Y.M. (2019). Current status, challenges and the way forward for dairy goat production in Europe. Asian-Australas. J. Anim. Sci..

[53] (2019). Anagrafe Nazionale Zootecnica. https://www.vetinfo.it/j6_statistiche/#/.

[54] ISMEA (2011). Analisi dei Regolamenti Comunali in Materia di Farmers’ Market.

[55] Barbera F., Dagnes J., Di Monaco R. (2018). Quality and Price Setting by Producers in AFNs. Alternative Food Networks.

[56] Garanti Z., Berberoglu A. (2018). Cultural Perspective of Traditional Cheese Consumption Practices and Its Sustainability among Post-Millennial Consumers. Sustainability.

[57] Kühl S., Gassler B., Spiller A. (2017). Labeling strategies to overcome the problem of niche markets for sustainable milk products: The example of pasture-raised milk. J. Dairy Sci..

[58] Mowlem A. (2005). Marketing goat dairy produce in the UK. Small Rumin. Res..

